# Phase II study of neoadjuvant checkpoint blockade in patients with surgically resectable undifferentiated pleomorphic sarcoma and dedifferentiated liposarcoma

**DOI:** 10.1186/s12885-018-4829-0

**Published:** 2018-09-24

**Authors:** Emily Z. Keung, Alexander J. Lazar, Keila E. Torres, Wei-Lien Wang, Janice N. Cormier, B. Ashleigh Guadagnolo, Andrew J. Bishop, Heather Lin, Kelly K. Hunt, Justin Bird, Valerae O. Lewis, Shreyaskumar R. Patel, Jennifer A. Wargo, Neeta Somaiah, Christina L. Roland

**Affiliations:** 1 Departments of Surgical Oncology, The University of Texas MD Anderson Cancer Center, 1400 Pressler St., FCT17.6054, Unit 1484, Houston, TX 77030 USA; 2 Departments of Pathology, The University of Texas MD Anderson Cancer Center, Houston, TX USA; 3 Departments of Genomic Medicine, The University of Texas MD Anderson Cancer Center, Houston, TX USA; 4 Departments of Radiation Oncology, The University of Texas MD Anderson Cancer Center, Houston, TX USA; 5 Departments of Biostatistics, The University of Texas MD Anderson Cancer Center, Houston, TX USA; 6 Departments of Orthopaedic Oncology, The University of Texas MD Anderson Cancer Center, Houston, TX USA; 7 Departments of Sarcoma Medical Oncology, The University of Texas MD Anderson Cancer Center, Houston, TX USA

**Keywords:** CTLA4, Immunotherapy, Ipilimumab, Liposarcoma, Neoadjuvant, Nivolumab, PD-1, Pembrolizumab, Soft tissue sarcoma, Undifferentiated pleomorphic sarcoma

## Abstract

**Background:**

Soft tissue sarcomas are a heterogeneous and rare group of solid tumors of mesenchymal origin that can arise anywhere in the body. Although surgical resection is the mainstay of treatment for patients with localized disease, disease recurrence is common and 5-year overall survival is poor (~ 65%). Both radiation therapy and conventional chemotherapy are used to reduce local and distant recurrence. However, the utility of radiation therapy is often limited by disease location (in the case of retroperitoneal sarcomas, for instance) while systemic therapy with conventional lines of chemotherapy offer limited efficacy and are often poorly tolerated and associated with significant toxicity. Within the past decade, major advances have been made in the treatment of other malignancies including melanoma, renal cell carcinoma, and non-small cell lung carcinoma with the advent of immune-checkpoint inhibitors such as ipilimumab (anti-CTLA4), pembrolizumab (anti-PD1), and nivolumab (anti-PD1). The recently published SARC028 (NCT02301039), an open label, phase II, multicenter trial of pembrolizumab in patients with advanced bone and soft tissue sarcomas reported promising activity in select histologic subtypes of advanced STS, including undifferentiated pleomorphic sarcoma and dedifferentiated liposarcoma.

**Methods:**

There is a clear need for novel and effective adjuncts in the treatment of STS. We hypothesize that immune checkpoint blockade will be effective in patients with surgically resectable primary or locally recurrent dedifferentiated liposarcoma and undifferentiated pleomorphic sarcoma when administered in the neoadjuvant setting. The primary aim of this phase II, single-center, open label, randomized non-comparative trial is to determine the pathologic response to neoadjuvant nivolumab monotherapy and combination nivolumab/ipilimumab in patients with resectable dedifferentiated liposarcoma of the retroperitoneum or undifferentiated pleomorphic sarcoma of the trunk or extremity treated with concurrent standard of care neoadjuvant radiation therapy.

**Discussion:**

This study will help define the role of single agent anti-PD1 and combination anti-CTLA4 and anti-PD1 therapy in patients with surgically resectable dedifferentiated liposarcoma and undifferentiated pleomorphic sarcoma.

**Trial registration:**

ClinicalTrials.gov NCT03307616, registered October 12, 2017.

**Electronic supplementary material:**

The online version of this article (10.1186/s12885-018-4829-0) contains supplementary material, which is available to authorized users.

## Background

Soft tissue sarcomas (STS) are a heterogeneous group of tumors originating from connective tissue with greater than 60 histologic subtypes [1, 2]. The mainstay of treatment for primary localized STS is surgical resection and achieving complete surgical resection is of critical importance as surgical resection margins have been shown to influence both local and distant recurrence-free survival as well as disease specific survival [3]. Despite optimal surgical resection, however, disease recurrence is common, with 5-year recurrence-free survival approaching 60% for some histologic subtypes, and overall survival remains poor [4].

Other treatment modalities, including radiation therapy and conventional chemotherapy, are used in the multidisciplinary management of STS. Radiation therapy has been shown to improve local disease control, whether given in the neoadjuvant or adjuvant setting, particularly in patients with large (> 5 cm), intermediate-grade or high-grade tumors, or in patients with uncertain or positive surgical margins [5–9]. Conventional cytotoxic chemotherapy remains the standard of care for the management of advanced sarcomas, and are limited by modest efficacy, considerable toxicity, and poor tolerability [10, 11].

With recent successes of immunotherapy across multiple solid tumors including melanoma, renal cell carcinoma, and non-small cell lung carcinoma, there has been increasing interest in applying immunotherapy to the treatment of STS [11–15]. These strategies are now being extended to patients with earlier stage disease across different histologies, with efforts being led at MD Anderson Cancer Center (NCT02231775). Compared to “immunogenic” tumors such as melanoma characterized by high mutation rates, STS are considered to be immunologically “quiet.” [16] Among STS subtypes, however, some histologies such as undifferentiated pleomorphic sarcoma (UPS) are characterized by complex genomic features [8], and a baseline immune infiltrate, which may provide immunologic mutated protein targets, and may respond to immune checkpoint inhibition [17, 18]. Indeed, this was described in the recently reported open-label phase II SARC028 trial (NCT02301039) of anti-PD-1 monotherapy in patients with advanced bone and soft tissue sarcomas [19]. In the SARC028 study, pembrolizumab demonstrated promising activity in UPS and dedifferentiated liposarcoma (DDLPS) with a 40% overall response rate in UPS and 20% in DDLPS.

As demonstrated in multiple tumor types, a significant subpopulation of patients treated with immunotherapy will not respond. At baseline, both the tumor immune microenvironment and the poor antigenicity of the tumor allow it to escape immune recognition. Radiation therapy can induce increased antigenic expression, release pro-inflammatory cytokines that recruit immune cells, promote antigen cross-presentation, and induce tumor expression of death receptors [20, 21]. Used together, radiation therapy and immunotherapy may have synergistic effects, which are being explored in lung and other cancers [22]. We have recently evaluated the immune infiltrate in a cohort of UPS patients treated with preoperative radiation. We found that at baseline there was a significant immune infiltrate present in the pretreatment biopsies and that, in general, there was an increase in the number of immune cells after irradiation [17]. We additionally found that at least focal hyalinization was present in 93% of post-treatment samples and that patients whose tumors demonstrated > 5% hyalinization following completion of neoadjuvant radiation therapy had better 3-year recurrence-free survival and a trend towards improved overall survival, suggesting that hyalinization could be used as a surrogate for outcomes. These are similar to results recently and previously reported by Schaefer et al. in their single institution experience of 100 patients with primary localized STS of the extremity or trunk treated with preoperative radiation therapy followed by surgical resection [23], suggesting that assessment of percent tumor hyalinization might be used as a readout of response to treatment.

There is an increasing appreciation of the role of the host microbiome in response to cancer therapy [24, 25] and evidence to suggest that bacteria present in the tumor [26] and the gut [25] may impact therapeutic responses. There is also a growing appreciation of the role of the gastrointestinal microbiome in shaping immune responses in health and disease [27, 28] and evidence that bacteria present within the gut may modulate differential responses to immune checkpoint blockade in melanoma by modulating T cell infiltrate in tumors [25]. This represents a significant translational knowledge gap, and insights gained could lead to therapeutic strategies to enhance responses to immune checkpoint blockade in sarcoma.

We have designed the following non-comparative, open-label, randomized phase II study to assess the pathologic response to nivolumab (anti-PD1) monotherapy and nivolumab in combination with ipilimumab (anti-CTLA4) administered in the neoadjuvant setting with and without concurrent neoadjuvant radiation in patients with immunotherapy-naïve primary or locally recurrent resectable undifferentiated pleomorphic sarcoma of the extremity/trunk (ET UPS) or dedifferentiated liposarcoma of the retroperitoneum (RP DDLPS).

## Methods/Design

### Study design

**Fig. 1:**
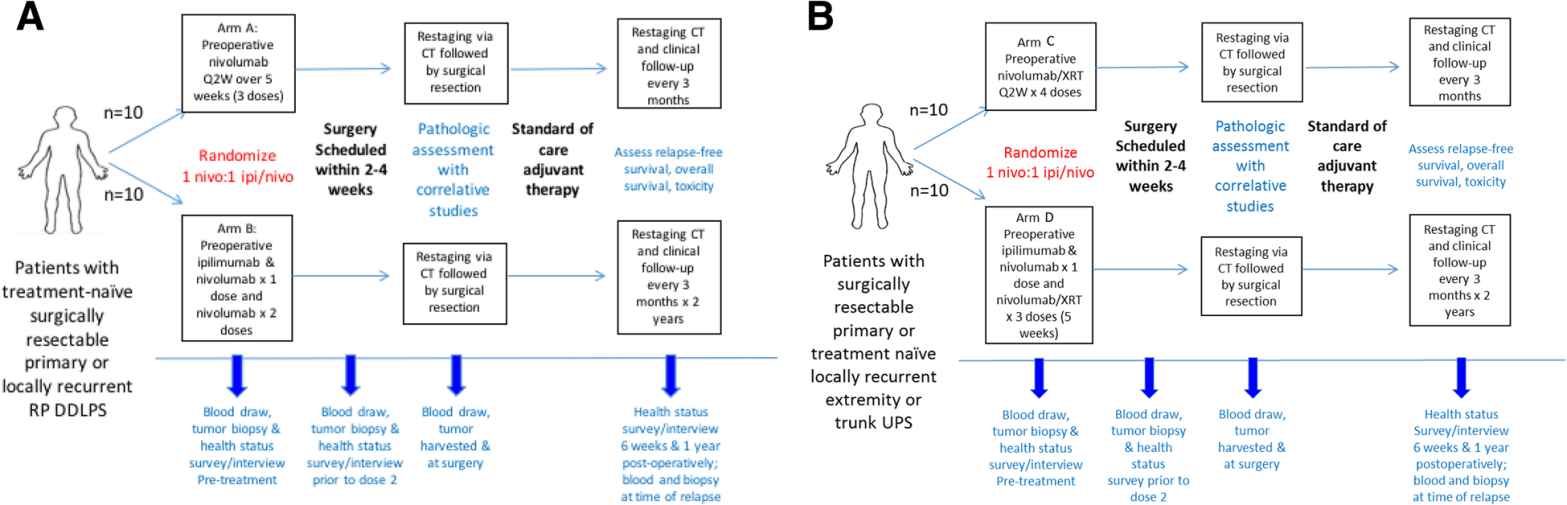
Study Schema. This is a randomized, non-comparative Phase II study designed to detect pathologic and immunologic biomarkers of response to checkpoint blockade in resectable, treatment naive primary or locally recurrent (**a**) dedifferentiated liposarcoma (DDLPS) of the retroperitoneum and (**b**) undifferentiated pleomorphic sarcoma (UPS) of the trunk or extremities

This is an investigator-initiated, single-center, randomized, open-label, phase II study to assess the treatment efficacy of nivolumab alone or in combination with ipilimumab in patients with surgically resectable soft tissue sarcoma (Fig. 1). Two cohorts of patients based on histology of the disease will be included in this trial. Each cohort will have two treatment arms:

#### Cohort 1: Dedifferentiated liposarcoma of the retroperitoneum (RP DDLPS)

Arm A: Neoadjuvant nivolumab monotherapy.

Arm B: Neoadjuvant nivolumb/ipilimumab combination therapy.

#### Cohort 2: Undifferentiated pleomorphic sarcomas (UPS) of the trunk or extremity (ET UPS)

Arm C: Neoadjuvant nivolumab monotherapy and concurrent neoadjuvant radiation therapy.

Arm D: Neoadjuvant nivolumb/ipilimumab combination therapy and concurrent neoadjuvant radiation therapy.

Twenty patients with treatment-naïve primary or locally recurrent RP DDLPS will be randomized in a 1:1 ratio. In arm A, 10 patients with treatment-naïve primary or recurrent RP DDLPS will receive 3 upfront doses of nivolumab (3 mg/kg every 2 weeks) prior to surgical resection. In arm B, 10 patients with treatment-naïve primary or recurrent RP DDLPS will receive 1 dose of ipilimumab (3 mg/kg on week 1) combined with nivolumab (1 mg/kg) followed by 2 doses of nivolumab (3 mg/kg every 2 weeks) prior to surgical resection.

As radiation therapy is standard of care for patients with sarcoma of the extremity/trunk, 20 patients with treatment-naïve primary or recurrent ET UPS will be randomized in a 1:1 ratio between arm C and arm D, both of which include combination nivolumab and concurrent XRT. In arm C, 10 ET UPS patients will receive 1 dose of nivolumub (3 mg/kg) followed by combination nivolumab (3 doses, 3 mg/kg every 2 weeks) plus radiation therapy. In arm D, 10 ET UPS patients will receive 1 dose of combination nivolumab (1 mg/kg) and ipilimumab (3 mg/kg), followed by combination nivolumab (3 doses, 3 mg/kg every 2 weeks) plus radiation therapy.

The primary objective is to assess pathologic response of DDLPS of the retroperitoneum and UPS of the trunk or extremity to neoadjuvant nivolumab monotherapy (arms A, C) and nivolumab/ipilimumab combination therapy (arm B, D) with and without radiation therapy. All participants must provide written informed consent. The trial received institutional review board approval on October 4, 2017 and is registered with ClinicalTrials.gov (NCT 03307616, registered October 12, 2017). The study schema is illustrated in Fig. 1 and the study calendars are available as Additional file 1.

### Trial organization and setting

The study is being conducted at The University of Texas MD Anderson Cancer Center (MDACC) with funding support provided by Bristol Myers Squibb. The funders will not participate in the study design, data collection, analyses and interpretation, or manuscript preparation. An independent data and safety monitoring board will monitor the conduct and safety of the trial to ensure patient safety. Stopping guidelines and monitoring practices have been established.

### Patient eligibility, selection, and randomization

Patients will be identified and approved for trial inclusion after a consensus panel of medical and surgical oncologists has determined that the disease is amenable to surgical resection and after the subject has passed screening evaluations. Eligible patients (Table 1) will be approached by a member of the research staff who will thoroughly explain the study and consent patients who choose to participate. Informed consent will be obtained by study personnel who will not be involved in the patient’s care.

**Table 1:** Key Eligibility Criteria

Key Inclusion Criteria	Key Exclusion Criteria
1. Adult subjects at least 18 years old with treatment-naïve primary or locally recurrent DDLPS of the retroperitoneum or UPS of the trunk/extremity	1. Active, known, or suspected autoimmune disease
2. Disease determined to be surgically resectable and are candidates for upfront surgery as agreed upon by multidisciplinary consensus	2. Known history of testing positive for human immunodeficiency virus or known acquired immunodeficiency syndrome
3. Recent imaging within 4 weeks of trial enrollment, demonstrating measure disease as defined by RECIST 1.1	3. Any positive test results for hepatitis B or C virus indicating acute or chronic infection
4. Must have at least 1 tumor amenable to serial biopsy in clinic or be willing to undergo serial biopsies through image-guided procedures during the neoadjuvant phase of the trial	4. Condition requiring systemic treatment with corticosteroids (> 10 mg daily prednisone equivalents) or other immunosuppressive medications within 14 days of study drug administration.
5. Must be medically fit to undergo surgery	5. Prior intraabdominal surgery within 4 weeks of trial enrollment
6. Must be immunotherapy-naïve	6. Prior chemotherapy for treatment of the sarcoma under consideration for resection or radiation therapy for sarcoma in the same area
7. Patients who have received prior conventional chemotherapy for another malignancy are eligible after a 28 day wash-out period	7. Active concurrent second malignancy
8. Patients must have organ/marrow function as defined below:	8. Current use of anticoagulants at therapeutic levels
a. WBC > 3 K/uL, ANC > 1 K/uL	
b. hemoglobin > 9 g/dL	
c. platelets > 1000 K/mm^3^	
d. serum creatinine < 2 mg/dL or creatinine clearance > 50 mL/min	
e. AST < 1.5 x upper limit of normal, ALT < 1.5 x upper limit of normal, bilirubin < 1.5 x upper limit of normal	

Enrolled patients will be assigned a subject number and subject randomization will be implemented by the Clinical Trial Conduct website maintained by the Department of Biostatistics at MDACC (https://biostatistics.mdanderson.org/ClinicalTrialConduct). Once a subject number has been assigned, it cannot be reassigned to any other patient. If the subject is prematurely discontinued from the study without having received the prescribed treatment, an additional subject may be enrolled as a replacement subject.

### Biospecimen collection and patient assessment

**Fig. 2:**
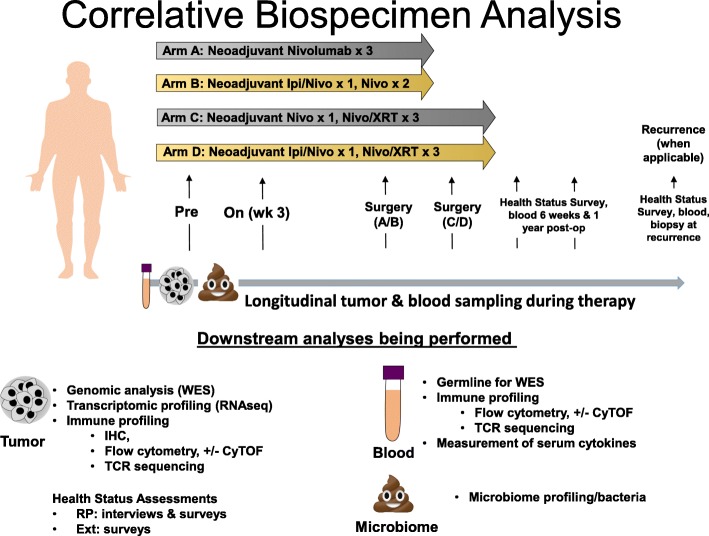
Correlative Biospecimen Collection and Analysis

Four sets of blood, tumor, and microbiome biospecimen collections are required: (1) at baseline (samples must be obtained prior to treatment initiation), (2) prior to dose 2 of neoadjuvant therapy in each study arm, (3) at the time of definitive surgical excision, and (4) at time of disease relapse or progression (Fig. 2, Additional file 1). An optional health status survey and microbiome specimen will also be administered at baseline and postoperatively at 6 weeks and 1 year after surgery as well as at disease recurrence or progression.

The primary objective of this study is assessment of percent tumor hyalinization as a marker of response to treatment at time of definitive surgical resection within each treatment arm [17, 23]. Histologic changes from baseline to on-treatment samples and the surgical specimen will also be assessed secondarily, including percentage of viable tumor cells, percent tumor necrosis, and amount of fibrosis and proliferation by phosphohistone H3. As is standard of care, restaging/surveillance imaging and clinical follow-up will be performed every 3 months postoperatively. See Additional file 1 for study calendars.

Secondary objectives of this study include assessment of immunologic changes in the tumor microenvironment and blood using tools such as immunohistochemistry (both singlet and multiplex) with image analysis and feature extraction, flow cytometry, and T-cell receptor (TCR) clonality studies to define the characteristics, patterns and molecular properties of the infiltrating tumor cells as well as PD-L1 expression by malignant cells. In addition, treatment response based on imaging in response to neoadjuvant therapy, toxicity, recurrence-free survival and overall survival will be determined. We will also assess the overall diversity and composition of the gut microbiome for association with response. Subjects will be assessed with computed tomography or magnetic resonance imaging at screening, after completion of neoadjuvant therapy and during the post-treatment follow-up period. Subjects will also be followed for survival for a minimum of two years. Additional genomic analyses may also be performed as sarcomas are rich in copy number alterations which have been correlated with immunotherapy response in melanoma [29].

Safety will be evaluated by clinical assessments including vital signs and complete physical examinations, chemistry and hematology laboratory values and formal assessments of adverse events, including immune related adverse events and delay to surgery.

### Statistics

A total of 40 patients will be enrolled. Twenty patients will be randomized in a 1:1 ratio between arm A and arm B in the RP DDLPS group and 20 patients will be randomized in a 1:1 ratio between arm C and arm D in the ET UPS group. The primary efficacy endpoint is pathologic response assessed at time of surgical resection by percentage hyalinization. With a sample size of 20 patients (10/arm) for each disease group, the trial will have 80% power to detect an effect size of 1.325. All patients randomized in the study will be included in the analysis in the arm assigned at randomization regardless of their adherence to entry criteria, treatment they actually received, compliance, or subsequent withdrawal from treatment or protocol deviation. We will estimate the difference of the pathologic response between the treatment arms (A vs B, C vs D) along with the estimate of variation of the difference. Given the longitudinal nature of the data, linear mixed effect models for longitudinal measures [30] will be employed to assess the change in the magnitude of the measures over time adjusting for multiple covariates including patient’s characteristics, and tumor characteristics. Appropriate transformation of the outcome assessment values will be used to satisfy the normality assumption of linear mixed effect model. We will also monitor grade 3 or 4 unacceptable toxicities related to drug within each study arm separately during the neoadjuvant period. The time window for toxicity monitoring is the entire neoadjuvant therapy period and 4 weeks after the completion of radiation therapy. If there is a high probability that the toxicity rate is likely to be greater than 30%, the regimen would not be considered of interest for further study.

## Discussion

Here we describe a single-institution phase II clinical trial to evaluate the role of checkpoint blockade in the neoadjuvant setting in patients with surgically resectable primary or recurrent retroperitoneal DDLPS and the role of checkpoint blockade with concurrent radiation therapy in the neoadjuvant setting in patients with surgically resectable primary or recurrent UPS of the extremity/trunk. This study, NCT 03307616, builds upon the recently reported results of the SARC028 trial that demonstrated clinical activity of anti-PD1 monotherapy in advanced unresectable DDLPS and UPS. In this study, patients with RP DDLPS will be randomized to receive either preoperative nivolumab (anti-PD1 monotherapy) or preoperative combination ipilimumab (anti-CTLA4) and nivolumab. Patients with ET UPS will be randomized to receive either preoperative nivolumab (anti-PD1 monotherapy) or preoperative combination ipilimumab (anti-CTLA4) and nivolumab, both with concomitant radiation therapy.

The primary efficacy endpoint is percent tumor hyalinization as a marker of response to treatment assessed at time of surgical resection. Secondary objectives of this study include treatment response based on imaging in response to neoadjuvant therapy, toxicity, recurrence-free survival and overall survival. Patients enrolled in this study will undergo serial collection of tumor, blood, and microbiome biospecimens at baseline, during immunotherapy treatment, at time of definitive surgery, and at time of disease recurrence or progression for histologic, genomic, immunologic, and microbiome analyses (Fig. 2).

Although the SARC028 trial demonstrated that anti-PD1 therapy has clinical activity in advanced UPS and DDLPS, a significant proportion of patients fail to respond to anti-PD1 monotherapy [19]. Using the biospecimens collected during this clinical trial, we will characterize molecular mechanisms of resistance to checkpoint blockade. Whole-exome sequencing of pretreatment and on-treatment specimens will be performed to assess for mechanisms of response and resistance (with a focus on anti-tumor immunity). These will be correlated with response (radiographic and pathologic) and survival endpoints.

The application of immune checkpoint blockade in sarcoma is in its infancy and we do not yet understand which patients will benefit from these therapies. However, in melanoma, recent deep molecular profiling of a cohort of longitudinal tissue samples from metastatic melanoma patients treated with sequential CTLA-4 blockade followed by PD-1 blockade demonstrated that a more clonal T cell repertoire was predictive of response to PD-1 blockade [29]. Analysis of copy number alterations also identified a higher burden of copy number loss in nonresponders to CTLA-4 and PD-1 blockade, particularly relevant in UPS and DDLPS where copy number alterations (particularly losses) are much more common relevant to point mutations [18]. Furthermore, the effect of mutational load and burden of copy number loss are likely nonredundant, suggesting the potential utility of a combinatorial biomarker. We thus hypothesize that in UPS and DDLPS molecular “signatures” exist in pre-treatment and early on-treatment biopsies in patients on checkpoint blockade that will be predictive of response. Resistance mechanisms may exist prior to treatment, or may emerge during therapy. Using rapid and cost-effective assays, we expect that correlative studies from this clinical trial will be hypothesis-generating and suggest potential novel approaches to modify treatment before significant escape occurs.

There is also early evidence that the gut and tumor microbiome may influence immune responses to cancer therapy [24, 26–28], though this has not been well studied in patients. Recently, differential bacterial “signatures” have been shown to be associated with response to checkpoint blockade in patients with melanoma [25]. Using fecal microbiome biospecimens collected during this study, we will test the hypothesis that differential microbiome signatures exist between UPS and DDLPS patients who respond to checkpoint blockade compared to those who fail to respond to immunotherapy.

In conclusion, we have designed the following non-comparative, open-label, randomized phase II to assess the pathologic response to nivolumab (anti-PD1) monotherapy and combination ipilimumab (anti-CTLA4)/nivolumab in the neoadjuvant setting with and without concurrent radiation therapy in patients with immunotherapy-naïve primary or locally recurrent resectable RP DDLPS and ET UPS. This study will help define the role of single agent anti-PD1 and combination anti-CTLA4 and anti-PD1 therapy in patients with surgically resectable DDLPS and UPS. Correlative biospecimen analyses will evaluate molecular mechanisms of resistance and determine predictive features of response to immune checkpoint blockade.

## Additional file

Additional file 1: Study Calendars. (DOCX 32 kb)

## Abbreviations

DDLPS: Dedifferentiated liposarcoma
ET UPS: Undifferentiated pleomorphic sarcoma of the extremity or trunk
MDACC: The University of Texas MD Anderson Cancer Center
RP DDLPS: Dedifferentiated liposarcoma of the retroperitoneum
STS: Soft tissue sarcoma
UPS: Undifferentiated pleomorphic sarcoma
