# Supplementary material for: Phase II study of neoadjuvant checkpoint blockade in patients with surgically resectable undifferentiated pleomorphic sarcoma and dedifferentiated liposarcoma
Source: BMC Cancer. 2018 Sep 24;18:913. doi: 10.1186/s12885-018-4829-0 (PMC6154892; doi:10.1186/s12885-018-4829-0)
Supplement: Supplementary file 1 — Study Calendars. (DOCX 32 kb) [file 12885_2018_4829_MOESM1_ESM.docx]

**Supplemental Materials:**

**Additional file 1: Table S1. Study Procedure Table for Patients Randomized to Arm A (neoadjuvant nivolumab)**

|  | Screening  28 days | Week 1 | Week 3 | Week 5 | Week 7 |
| --- | --- | --- | --- | --- | --- |
|  |  | Day 1 ^h^ | Day 15^i^ | Day 29 ^j^ | Days 43^k^ |
| Informed consent | X |  |  |  |  |
| Demographics | X |  |  |  |  |
| Medical history | X |  |  |  |  |
| Concurrent medications | X | X | X | X | X |
| AEs | X | X | X | X | X |
| PE/Vitals/ECOG^a^ | X | X | X | X | X |
| Pregnancy test^b^ | X |  |  |  | X |
| CBC with diff | X | X | X | X | X |
| Serum chemistry^c^ | X | X | X | X | X |
| TSH, free T4, free T3 | X | X | X | X |  |
| Coagulation | X |  |  |  | X |
| Hepatitis B and C | X |  |  |  |  |
| 12-lead EKG | X |  |  |  | X |
| Biopsy^d^ | X |  | X |  |  |
| Research Blood^e^ | X |  | X |  | X |
| Optional Microbiome assessment | X |  | X |  | X |
| Optional Health Status Assessment | X |  | X |  | X |
| Disease Assessment^f^ | X |  |  |  | X |
| Surgical resection^g^ |  |  |  |  | X |
| Nivolumab 3mg/kg |  | X | X | X |  |

1. Systolic and diastolic blood pressure, respiratory rate, pulse rate, and temperature. Must include full skin examination.
2. Serum or urine β-hCG - For women of childbearing potential only within 7 days of start of study drugs. If you become pregnant, a serum or urine β-hCG will be done to confirm pregnancy
3. Albumin, alkaline phosphatase, total bilirubin, bicarbonate, BUN, calcium, chloride, creatinine, glucose, , magnesium, phosphorus, potassium, total protein, AST, ALT, sodium, CPK
4. Minimal 5mm core biopsy of safely accessible lesion. Baseline biopsies are required for all enrolled.
5. 30cc of blood will be obtained at each time point for immunologic and genomic correlative studies
6. CT of the chest, abdomen, pelvis
7. Surgical resection of residual tumor if restaging scans show disease stability, favorable response to treatment, or progression of disease that is still deemed surgically resectable. A sample of the tumor will be obtained at the time of surgery to test for any residual sarcoma cells and/or progression of disease.
8. Day 1 +/- 3 days
9. Day 15 +/- 3 days
10. Day 29 +/- 3 days
11. Day 43 +/- 3 days

**Additional file 1: Table S2. Study Procedure Table for Patients Randomized to Arm B (neoadjuvant nivolumab/ipilimumab)**

|  | Screening  28 days | Week 1 | Week 3 | Week 5 | Week 7 |
| --- | --- | --- | --- | --- | --- |
|  |  | Day 1^h^ | Day 15^i^ | Day 29^j^ | Days 43^k^ |
| Informed consent | X |  |  |  |  |
| Demographics | X |  |  |  |  |
| Medical history | X |  |  |  |  |
| Concurrent medications | X | X | X | X | X |
| AEs | X | X | X | X | X |
| PE/Vitals/ECOG^a^ | X | X | X | X | X |
| Pregnancy test^b^ | X |  |  |  | X |
| CBC with diff | X | X | X | X | X |
| Serum chemistry^c^ | X | X | X | X | X |
| TSH, free T4, free T3 | X | X | X | X |  |
| Coagulation | X |  |  |  | X |
| Hepatitis B and C | X |  |  |  |  |
| 12-lead EKG | X |  |  |  | X |
| Biopsy^d^ | X |  | X |  |  |
| Research Blood^e^ | X |  | X |  | X |
| Optional Microbiome assessment | X |  | X |  | X |
| Optional Health Status Assessment | X |  | X |  | X |
| Disease Assessment^f^ | X |  |  |  | X |
| Surgical resection^g^ |  |  |  |  | X |
| Nivolumab 1mg/kg + ipilimumab 3mg/kg |  | X |  |  |  |
| Nivolumab 3mg/kg |  |  | X | X |  |

1. Systolic and diastolic blood pressure, respiratory rate, pulse rate, and temperature. Must include full skin examination.
2. Serum or urine β-hCG - For women of childbearing potential only within 7 days of start of study drugs. If you become pregnant, serum or urine β-hCG will be performed to confirm pregnancy.
3. Albumin, alkaline phosphatase, total bilirubin, bicarbonate, BUN, calcium, chloride, creatinine, glucose, , magnesium, phosphorus, potassium, total protein, AST, ALT, sodium, CPK
4. Minimal 5mm core biopsy of safely accessible lesion. Baseline biopsies are required for all enrolled patients.
5. 30cc of blood will be obtained at each time point for immunologic and genomic correlative studies
6. CT of the chest, abdomen, pelvis
7. Surgical resection of residual tumor if restaging scans show disease stability, favorable response to treatment, or progression of disease that is still deemed surgically resectable. A sample of the tumor will be obtained at the time of surgery to test for any residual sarcoma cells and/or progression of disease.
8. Day 1 +/- 3 days
9. Day 15 +/- 3 days
10. Day 29 +/- 3 days
11. Day 43 +/-3 days

**Additional file 1: Table S3. Study Procedure Table for Patients Randomized to Arm C (neoadjuvant nivolumab)**

|  | Screening | Week 1 | Week 3 | Week 5 | Week 7 | Week 11 |
| --- | --- | --- | --- | --- | --- | --- |
|  |  | Day 1^i^ | Day 15^j^ | Day 29^k^ | Day 43^l^ | Days 7m |
| Informed consent | X |  |  |  |  |  |
| Demographics | X |  |  |  |  |  |
| Medical history | X |  |  |  |  |  |
| Concurrent medications | X | X | X | X | X | X |
| AEs | X | X | X | X | X | X |
| PE/Vitals/ECOG^a^ | X | X | X | X | X | X |
| Pregnancy test^b^ | X |  |  |  |  | X |
| CBC with diff | X | X | X | X | X | X |
| Serum chemistry^c^ | X | X | X | X | X | X |
| TSH, free T4, free T3 | X | X | X | X | X |  |
| Coagulation | X |  |  |  |  | X |
| Hepatitis B and C | X |  |  |  |  |  |
| 12-lead EKG | X |  |  |  |  | X |
| Biopsy^d^ | X |  | X |  |  |  |
| Research Blood^e^ | X |  | X |  |  | X |
| Optional Microbiome assessment | X |  | X |  |  | X |
| Optional Health Status Assessment^n^ | X |  | X |  |  | X |
| Disease Assessment^f^ | X |  |  |  |  | X |
| Surgical resection^g^ |  |  |  |  |  | X |
| Nivolumab 3mg/kg |  | X | X | X | X |  |
| XRT^h^ |  |  | X | X | X |  |

1. Systolic and diastolic blood pressure, respiratory rate, pulse rate, and temperature. Must include full skin examination.
2. Serum or urine β-hCG - For women of childbearing potential only within 7 days of start of study drugs. If you become pregnant, serum or urine β-hCG will be used to confirm pregnancy.
3. Albumin, alkaline phosphatase, total bilirubin, bicarbonate, BUN, calcium, chloride, creatinine, glucose, , magnesium, phosphorus, potassium, total protein, AST, ALT, sodium, CPK
4. Minimal 5mm core biopsy of safely accessible lesion. Baseline biopsies are required for all enrolled.
5. 30cc of blood will be obtained at each time point for immunologic and genomic correlative studies
6. CT of the chest, and MRI of the extremity/trunk
7. Surgical resection of residual tumor if restaging scans show disease stability, favorable response to treatment, or progression of disease that is still deemed surgically resectable. A sample of the tumor will be obtained at the time of surgery to test for any residual sarcoma cells and/or progression of disease.
8. 5000cGy over 25 fractions
9. Day 1 +/- 3 days
10. Day 15 +/- 3 days
11. Day 29 +/- 3 days
12. Day 43 +/- 3 days
13. Day 71 + 14 days or - 7 days
14. Health status instrument only-no interview

**Additional file 1: Table S4. Study Procedure Table for Patients Randomized to Arm D (neoadjuvant nivolumab/ ipilimumab)**

|  | Screening | Week 1 | Week 3 | Week 5 | Week 7 | Week 11 |
| --- | --- | --- | --- | --- | --- | --- |
|  |  | Day 1^i^ | Day 15^j^ | Day 29^k^ | Day 43^l^ | Days 71^m^ |
| Informed consent | X |  |  |  |  |  |
| Demographics | X |  |  |  |  |  |
| Medical history | X |  |  |  |  |  |
| Concurrent medications | X | X | X | X | X | X |
| AEs | X | X | X | X | X | X |
| PE/Vitals/ECOG^a^ | X | X | X | X | X | X |
| Pregnancy test^b^ | X |  |  |  |  | X |
| CBC with diff | X | X | X | X | X | X |
| Serum chemistry^c^ | X | X | X | X | X | X |
| TSH, free T4, free T3 | X | X | X | X | X |  |
| Coagulation | X |  |  |  |  | X |
| Hepatitis B and C | X |  |  |  |  |  |
| 12-lead EKG | X |  |  |  |  | X |
| Biopsy^d^ | X |  | X |  |  |  |
| Research Blood^e^ | X |  | X |  |  | X |
| Optional Microbiome assessment | X |  | X |  |  | X |
| Optional Health Status Assessment^n^ | X |  | X |  |  | X |
| Disease Assessment^f^ | X |  |  |  |  | X |
| Surgical resection^g^ |  |  |  |  |  | X |
| Nivolumab 1mg/kg + ipilimumab 3mg/kg |  | X |  |  |  |  |
| Nivolumab 3mg/kg |  |  | X | X | X |  |
| XRT^h^ |  |  | X | X | X |  |

1. Systolic and diastolic blood pressure, respiratory rate, pulse rate, and temperature. Must include full skin examination.
2. Serum or urine β-hCG - For women of childbearing potential only within 7 days of start of study drugs. If you become pregnant, serum or urine β-hCG will be used to confirm pregnancy.
3. Albumin, alkaline phosphatase, total bilirubin, bicarbonate, BUN, calcium, chloride, creatinine, glucose, , magnesium, phosphorus, potassium, total protein, AST, ALT, sodium, CPK
4. Minimal 5mm core biopsy of safely accessible lesion. Baseline biopsies are required for all enrolled.
5. 30cc of blood will be obtained at each time point for immunologic and genomic correlative studies
6. CT of the chestand MRI of the extremity/trunk
7. Surgical resection of residual tumor if restaging scans show disease stability, favorable response to treatment, or progression of disease that is still deemed surgically resectable. A sample of the tumor will be obtained at the time of surgery to test for any residual sarcoma cells and/or progression of disease.
8. 5000cGy over 25 fractions
9. Day 1 +/- 3 days
10. Day 15 +/- 3 days
11. Day 29 +/- 3 days
12. Day 43 +/- 3 days
13. Day 71 + 14 days or - 7 days
14. Health status instrument only-no interview

**Additional file 1: Table S5: Post-operative Follow-up schedule for all patients**

|  | Post-op Week 6^g^ | Post-op Week 18^h^ | Post-op Week 30^i^ | Post-op Week 42^j^ | Post-op Week 54^k^ | Post-op Week 66^l^ | Post-op Week 78^m^ | Post-op Week 90^n^ | Post-op Week 102^o^ |
| --- | --- | --- | --- | --- | --- | --- | --- | --- | --- |
| Demographics | X | X | X | X | X | X | X | X | X |
| Medical history | X | X | X | X | X | X | X | X | X |
| Concurrent medications | X | X | X | X | X | X | X | X | X |
| AEs | X | X | X | X | X | X | X | X | X |
| PE/Vitals/ECOG^a^ | X | X | X | X | X | X | X | X | X |
| CBC with diff | X |  |  |  | X |  |  |  | X |
| Serum chemistry^b^ | X |  |  |  | X |  |  |  | X |
| Biopsy^c^ |  | * | * | * | * | * | * | * | * |
| Optional Microbiome assessment |  | * | * | * | * | * | * | * | * |
| Research Blood^d^ | X | * | * | * | X | * | * | * | * |
| Disease Assessment^e^ | X | X | X | X | X | X | X | X | X |
| Optional Health Status Assessment^f^ | X | * | * | * | X | * | * | * | * |

1. Systolic and diastolic blood pressure, respiratory rate, pulse rate, and temperature. Must include full skin examination.
2. Albumin, alkaline phosphatase, total bilirubin, bicarbonate, BUN, calcium, chloride, creatinine, glucose, , magnesium, phosphorus, potassium, total protein, AST, ALT, sodium
3. Minimal 5mm core biopsy of safely accessible lesion. *Biopsies, and research blood at recurrence/progression are required for all enrolled. Optional health status survey and microbiome assessment.
4. 30cc of blood will be obtained at each time point for immunologic and genomic correlative studies
5. CT of the chest, abdomen, pelvis, or extremity as clinically indicated (or MRI vs PET CT)
6. Health status assessment (All groups); Qualitative health-status interview (Arm A & B only).
7. Week 6 +/- 14 days
8. Week 18 +/- 28 days
9. Week 30 +/- 28 days
10. Week 42 +/- 28 days
11. Week 54 +/- 28 days
12. Week 66 +/- 28 days
13. Week 78 +/- 28 days
14. Week 90 +/- 28 days
15. Week 102 +/- 28 days
